# Cannabis Use Increases the Risk of Sickness Absence: Longitudinal Analyses From the CONSTANCES Cohort

**DOI:** 10.3389/fpubh.2022.869051

**Published:** 2022-05-30

**Authors:** Amélia Déguilhem, Annette Leclerc, Marcel Goldberg, Cédric Lemogne, Yves Roquelaure, Marie Zins, Guillaume Airagnes

**Affiliations:** ^1^INSERM, Population-based Epidemiological Cohorts Unit, UMS 011, Villejuif, France; ^2^Université Paris Cité, AP-HP, Hôpital Hôtel-Dieu, DMU Psychiatrie et Addictologie, Service de Psychiatrie de l'adulte, INSERM, Institute of Psychiatry and Neuroscience of Paris, UMR_S1266, Paris, France; ^3^Centre Hospitalier Universitaire d'Angers, Pathologie Professionnelle et Médecine du Travail, Research Institute for Environmental and Occupational Health, INSERM, Ester, Epidemiology in Occupational Health and Ergonomics, UMR_S 1085, Angers, France; ^4^Université Paris Cité, AP-HP, Hôpital Européen Georges Pompidou, DMU Psychiatrie et Addictologie, Centre Ambulatoire d'Addictologie, INSERM, Population-based Epidemiological Cohorts Unit, UMS 011, Villejuif, France

**Keywords:** cannabis, sickness absence, sick leave, occupational health, work

## Abstract

**Aims:**

To examine the longitudinal associations between cannabis use and risks of short (<7 days), medium (7-28 days), and long (>28 days) sickness absences at one-year follow-up.

**Methods:**

87,273 participants aged 18-65 years from the French CONSTANCES cohort reported their frequency of cannabis use at inclusion between 2012 and 2018. Sickness absences occurring during one year of follow-up were collected from national medico-administrative registries. Multivariable generalized linear regressions were used to compute the Odds Ratios (OR) with their 95% Confidence Intervals (CI) of having at least one sickness absence at follow-up compared to no sickness absence, while controlling for sociodemographic factors, chronic conditions and occupational factors.

**Results:**

Cannabis use more than once a month was associated with an increased risk of short (OR, [95% CI]: 1.56 [1.32–1.83]) and medium (1.29 [1.07–1.54]) sickness absences at one-year follow-up, with dose-dependent relationships for short sickness absences (1.13 [1.08–1.18], *p*-for-trend <0.001). In stratified analyses, cannabis use was associated with an increased risk of sickness absences in older individuals, men, participants with good self-rated health, living or having lived as a couple, and having an open-ended contract.

**Conclusions:**

Cannabis use prospectively increased the risk of short and medium sickness absences, even from once a month and with a dose-dependent relationship for short sickness absences. These findings should be considered in information and prevention public health campaigns to alert the general population and workers to this increased risk.

## Highlights

- Short and medium sickness absences increase when consuming cannabis regularly.
- Dose-dependent relationships were found regarding short sickness absences.
- Prevention campaigns must be updated to better inform on the harm of cannabis use.

## Introduction

Cannabis is the most widely consumed illicit substance in the world, with a higher prevalence in men (1, 2). Cannabis use is associated with several detrimental health consequences including psychiatric (e.g., mood and anxiety disorders, acute psychotic episode) and somatic disorders (e.g., cardiovascular, respiratory and other diseases) (1). With around 700,000 daily users, France ranks first out of 30 European countries regarding the prevalence of cannabis use in the general population (3). Its use in the adult population has recently spread to groups that were not previously the most concerned, particularly the working population (4). In France, the reported past-year cannabis consumption tripled (from 3.5 to 9.6%) between 1992 and 2017 among the employed population (5). Among workers, the prevalence of cannabis use may vary according to sociodemographic and occupational factors. The emergence of movements in favor of decriminalization or liberalization of cannabis for medical and/or recreative purposes could increase the use of this substance further, and especially among workers (6, 7).

In addition to detrimental health consequences, cannabis use could also have deleterious social and occupational consequences. Indeed, both acute and long-term effects of cannabis use may alter occupational life, at least through the alteration of interpersonal relationships promoted by cannabis-induced psychiatric disorders. These effects might even lead to job loss whereas substance users could be more affected by socioeconomic crises than the general population (8). Furthermore, even if employed people are rather less prone to addictive behaviors compared to unemployed ones, exposure to certain occupational factors may be associated with increased cannabis use (5, 9). Another negative consequence of cannabis use on working life may be an increased risk of sickness absences. Measuring the frequency and duration of sickness absences could be a good way to assess the role of cannabis in working life. Indeed, sickness absences are a good indicator of the overall impact of impaired health on working life (10). Sickness absences are associated with an increase in morbidity and mortality, and when repeated and/or prolonged, they are associated with the development of unhealthy behaviors, such as the use of psychoactive substances, a sedentary lifestyle and poor eating habits (11). In addition, the occurrence of sickness absences is also associated with an increased risk of job loss and unemployment, as well as with major medico-economic consequences (12). Although longitudinal associations have been described between alcohol and tobacco use and the risk of sickness absences (13, 14), these associations have been much less studied for cannabis use. To our knowledge, no study to date has examined the prospective associations between cannabis use and the risk of sickness absences. Nevertheless, the study of associations between cannabis use and sickness absences requires taking into account potential confounding and/or moderating factors, whether clinical or sociodemographic.

The French CONSTANCES cohort offers a unique opportunity to examine the prospective relationships between cannabis use and the risk of sickness absence while taking into account a broad range of sociodemographic factors, chronic conditions and occupational factors. Indeed, CONSTANCES is a national population-based cohort including a randomized sample of the French population from various occupational status types and sociodemographic factors (15). In addition, CONSTANCES benefits from a linkage with administrative medical registries providing an objective and exhaustive assessment of health care consumptions, including sickness absences (16). We hypothesized that frequency of cannabis use will be positively associated with subsequent risk of sickness absence with dose-dependent relationships.

## Methods

### Participants

CONSTANCES is a French national population-based cohort that included more than 220,000 volunteers living in metropolitan France and aged 18 to 69 years at inclusion (15). Participants were selected from French adults covered by the National Health Insurance Fund according to a random sampling scheme stratified on age, gender, socioeconomic status and region of France. All volunteers receive a health check-up at inclusion and answer annual questionnaires with information concerning, in particular, their socio-demographic and socio-professional situation, their health status and their lifestyle.

**Figure 1:**
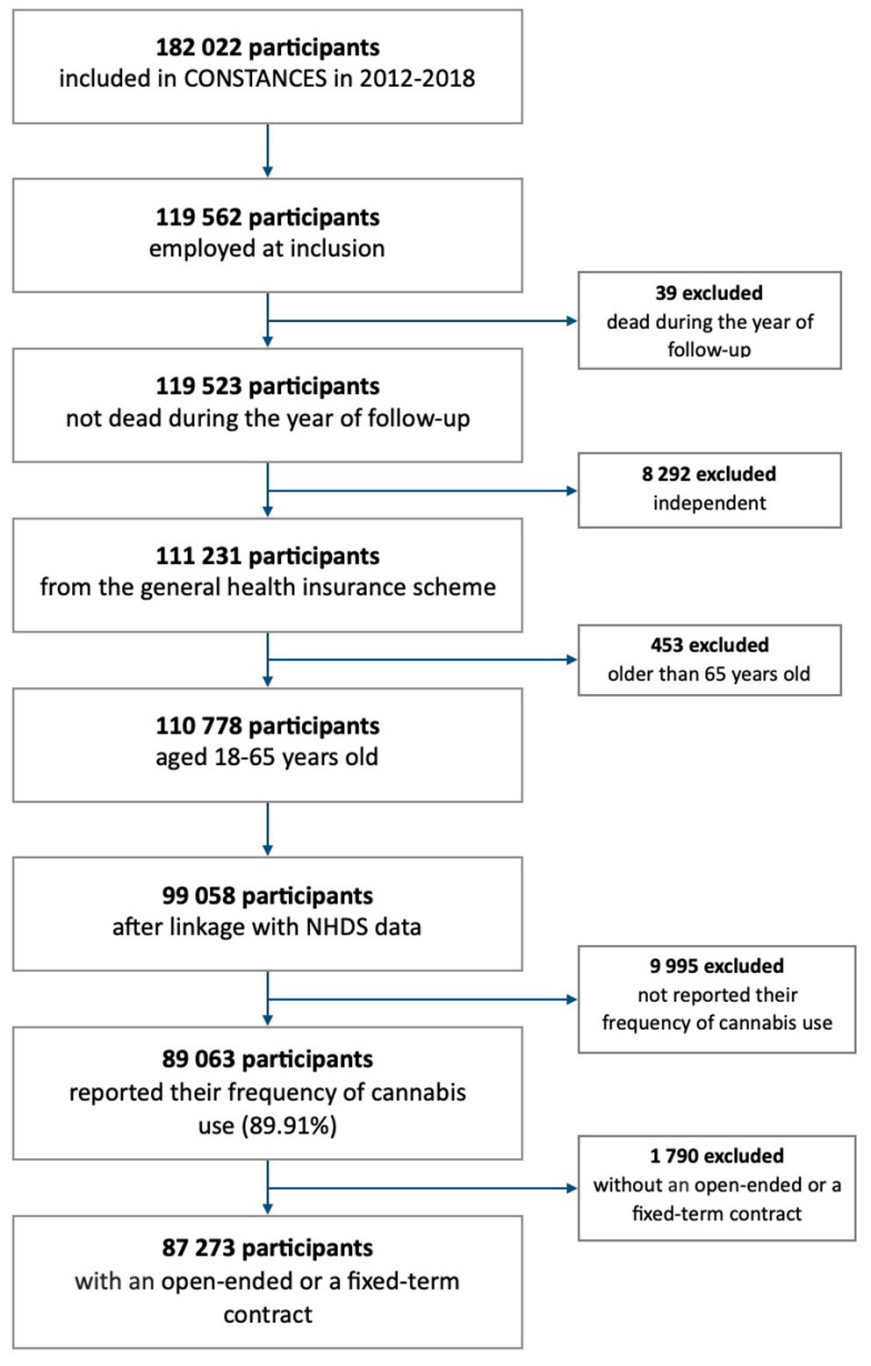
Flow-chart of the study population.

Among the 182,022 volunteers included from 2012 to 2018, 119,562 participants were employed at inclusion. After excluding 8,292 independent workers and 453 participants older than 65 years, we made the linkage with the National Health Data System—NHDS (Système National des Données de Santé—SNDS) that was feasible for 99,058 participants. Among them, 89,063 (89.9%) reported their cannabis use at inclusion. Finally, information regarding the type of employment contract (i.e., open-ended contract or fixed-term contract) was provided for 87,273 participants (98.0%). All of these participants were included in the present study (Figure 1).

The CONSTANCES cohort has obtained the authorization of the National Data Protection Authority (*Commission Nationale de l*'*Informatique et des Libertés—CNIL*, no. 910486) and was approved by the Institutional Review Board of the National Institute for Medical Research—*INSERM* (no. 01–011). Written informed consent was received from all subjects in the cohort.

### Cannabis Use

Participants were asked at baseline if they had ever taken cannabis. Those who have ever used cannabis were asked to characterize the frequency of their cannabis use with the following questions: “How many times have you taken it over the past 12 months?” and “How many times have you taken it over the past 30 days?”. Based on this information, we computed a categorical variable measuring the frequency of cannabis consumption as follows: (1) never used, (2) no use in the last 12 months, (3) less than once a month in the last 12 months, (4) once a month or more in the last 12 months.

### Sickness Absences

The systematic linkage of the CONSTANCES cohort to the National Health Data System—NHDS (*Système National des Données de Santé—SNDS*) provides access to objective and exhaustive data regarding duration and date of occurrence of sickness absences, from January 2009 to December 2019 at the time of study (16). Based on this information, we computed the number of short (<7 days), medium (from 7 to 28 days) and long (>28 days) sickness absences that occurred over one year of follow-up since baseline.

### Covariables

From the baseline questionnaire, we used age in years that has been categorized (≥18 and ≤35; >35 and ≤50; >50 and ≤65), gender (men or women), marital status (single; married or in a civil partnership; separated, divorced or widowed), household income in euros per month (<1,500; 1,500–4,200; >4,200) and occupational grade (blue-collar worker and clerk; intermediate worker; executive), as categorical variables. Education level was based on the International Standard Classification of Education as follows: levels 0 to 4 (up to upper secondary education or post-secondary non-tertiary education), levels 5 and 6 (short-cycle tertiary education and Bachelor's or equivalent level), and levels 7 and 8 (Master's or equivalent level and Doctoral or equivalent level) (17). Tobacco consumption was used as a continuous variable counting the lifetime number of pack-years, with non-smokers having zero pack-years. Self-rated health status was measured from the following question: “How do you judge your general health compared to a person of your entourage of the same age?” to provide a proxy of overall physical and psychological health condition. We computed a binary variable to define a good general health (score 1–4) vs. a poor one (score 5–8) (18). History of treated depression and current long-term condition were queried and used as binary variables (“Yes“ or “No“). Occupational factors as type of contract (i.e., open-ended or fixed-term) and constraints at work (i.e., work stress and stressful public exposure) were also assessed. Work stress was measured by the French version of the short Effort–Reward Imbalance (ERI) Questionnaire (19). A ratio >1 was used to identify participants with ERI in favor of efforts from those with no ERI or ERI in favor of rewards (20). Among these participants with ERI, we defined the most stressed as those in approximately the highest decile, which corresponded to a ratio >1.5. Thus, work stress was categorized in three levels as follows: light (<1), moderate (from 1 to 1.5) and heavy (>1.5). Stressful public exposure was computed from two questions: “Are you in physical or phone contact with the public (users, patients, travelers, customers, etc.) every day, or nearly?” and “If yes, do you experience tense situations in your relations with the public?” to obtain three categories: no contact with the public, contact without tension and contact with tension.

### Statistical Analysis

Three different dependent variables were considered for all the analyses, i.e., the number of short, medium and long sickness absences at one-year of follow-up. In the main analyses, these dependent variables were used as binary outcomes, i.e., no sickness absence vs. at least one sickness absence. The independent variable (i.e., the frequency of cannabis use) was used as an ordered categorical variable in the main analysis.

Univariable generalized linear regression models were used to study the risk of sickness absence at one year according to the frequency of cannabis use at baseline. The results were presented as Odds Ratios (OR) with their 95% Confidence Intervals (CI). Multivariable generalized linear regression models were then performed by introducing in the models the following covariables: sociodemographic, chronic conditions and occupational factors. Three levels of adjustment were performed. Since age and gender are strongly linked to both cannabis use and sickness absences, the first level of adjustment was for age and gender. Then, the second one was additionally adjusted for tobacco consumption in order to study the specific role of cannabis and not tobacco as it is frequently associated. Finally, the last level of adjustment included also all the other potential covariables in order to examine their potential confounding and/or moderating roles. Covariables were studied as potential moderators by testing their interactions with cannabis use in order to identify at-risk subgroups. In case of significant interaction, stratified analyses were performed. Stratified analyses for age, gender and smoking status were conducted whatever the significance of the interactions between cannabis use and these variables, as the magnitude of the associations between cannabis and sickness absences may differ between different strata of the population. Finally, we searched for dose-dependent relationships between the frequency of cannabis use and the risk of sickness absence by introducing the frequency of cannabis use as a continuous variable in the models instead of introducing it as a categorical variable in order to compute a *p* for trend.

In sensitivity analyses, stratification for sickness absences in the 3 years before inclusion, i.e., zero sickness absence vs. at least one sickness absence, was performed. We also examined whether the associations could remain similar while performing a negative binomial regression with count data for the dependent variables.

Non-response biases were examined by comparing participants who reported their cannabis use at baseline with those who did not.

We had complete data for the outcomes (i.e., sickness absences) since data from the National Health Data System (NHDS) are exhaustively registered. We didn't have missing data for the independent variable of interest as we selected only the participants who reported their frequency of cannabis use. Regarding the covariables, we had missing data with a mean percentage of 6.1%. Assuming that these data were randomly missing, multiple imputations were preferred to restricted analysis to complete data in order to limit selection biases (21).

Statistical significance was determined using a conservative two-sided alpha a priori set at 0.05 and statistical analyses were performed with RStudio, version 1.3 (R foundation).

## Results

### Characteristics of the Participants

Among the 87,273 participants, 14,219 (16.3%) experienced at least one sickness absence at one-year of follow-up. The majority of these participants (*n* = 8,448; 59.4%) never used cannabis, more than a third (*n* = 5212; 36.7%) experienced cannabis more than one year ago, 219 (1.5%) used cannabis less than once a month in the last 12 months and 340 (2.4%) used cannabis once a month or more in the last 12 months.

Sickness absences were more prevalent among women, younger, and people with poorer sociodemographic factors. All the participants' characteristics are displayed in Table 1.

**Table 1:** Characteristics of the participants according to sickness absence at follow-up whatever their duration.

	**No sickness absence**	**At least one sickness absence**	**Total**
	**(*N* = 73,054)**	**(*N* = 14,219)**	**(*N* = 87,273)**
**Gender**			
Men	33,678 (46.1%)	6,084 (42.8%)	39,762 (45.6%)
Women	39,376 (53.9%)	8,135 (57.2%)	47,511 (54.4%)
**Age**			
Median [Min, Max]	43.5 [18.5, 65.0]	42.5 [18.5, 65.0]	43.5 [18.5, 65.0]
[18,35]	17,404 (23.8%)	3,957 (27.8%)	21,361 (24.5%)
[35,50]	34,676 (47.5%)	6,324 (44.5%)	41,000 (47.0%)
[50,65]	20,974 (28.7%)	3,938 (27.7%)	24,912 (28.5%)
**Frequency of cannabis use**			
Never used	44,092 (60.4%)	8,448 (59.4%)	52,540 (60.2%)
Prior use more than one year ago	26,763 (36.6%)	5,212 (36.7%)	31,975 (36.6%)
Less than once a month	1,053 (1.4%)	219 (1.5%)	1,272 (1.5%)
More than once a month	1,146 (1.6%)	340 (2.4%)	1,486 (1.7%)
**Marital status**			
Single	19,865 (27.2%)	4,086 (28.7%)	23,951 (27.4%)
Married or in a civil partnership	45,649 (62.5%)	8,418 (59.2%)	54,067 (62.0%)
Separated, divorced or widowed	7,540 (10.3%)	1,715 (12.1%)	9,255 (10.6%)
**Occupational grade**			
Blue collar worker and clerk	22,283 (30.5%)	7,640 (53.7%)	29,923 (34.3%)
Intermediate worker	23,638 (32.4%)	3,081 (21.7%)	26,719 (30.6%)
Executive	27,133 (37.1%)	3,498 (24.6%)	30,631 (35.1%)
**Income**			
<1,500 €/month	4,132 (5.7%)	1,466 (10.3%)	5,598 (6.4%)
1,500–4,200 €/month	42,751 (58.5%)	9,706 (68.3%)	52,457 (60.1%)
>4,200 €/month	26,171 (35.8%)	3,047 (21.4%)	29,218 (33.5%)
**Education**			
ISCED—levels 0–4	21,133 (28.9%)	6,778 (47.7%)	27,911 (32.0%)
ISCED—levels 5-6	29,266 (40.1%)	4,810 (33.8%)	34,076 (39.0%)
ISCED—levels 7-8	22,655 (31.0%)	2,631 (18.5%)	25,286 (29.0%)
**Number of pack-years (10 PY)**			
Median [Min, Max]	0 [0, 120]	1 [0, 120]	0 [0, 120]
**Self-rated health**			
Good	66,157 (90.6%)	11,586 (81.5%)	77,743 (89.1%)
Bad	6,897 (9.4%)	2,633 (18.5%)	9,530 (10.9%)
**History of depression**			
No	63,818 (87.4%)	11,495 (80.8%)	75,313 (86.3%)
Yes	9,236 (12.6%)	2,724 (19.2%)	11,960 (13.7%)
**Chronic condition**			
No	70,370 (96.3%)	2,795 (81.7%)	2,795 (81.7%)
Yes	2,684 (3.7%)	626 (18.3%)	626 (18.3%)
**Work contract**			
Open-ended	3,298 (96.4%)	13,335 (93.8%)	82,374 (95.9%)
Fixed-term	123 (3.6%)	884 (6.2%)	4,899 (5.6%)
**Work stress**			
Light	38,598 (52.8%)	6,159 (43.3%)	44,757 (51.3%)
Moderate	25,769 (35.3%)	5,222 (36.7%)	30,991 (35.5%)
Heavy	8,687 (11.9%)	2,838 (20.0%)	11,525 (13.2%)
**Stressful exposure to the public**			
No exposure	21,378 (29.3%)	4,429 (31.1%)	25,807 (29.6%)
No stressful exposure	36,868 (50.5%)	6,360 (44.7%)	43,228 (49.5%)
Stressful exposure	14,808 (20.3%)	3,430 (24.1%)	18,238 (20.9%)

*ISCED, International Standard Classification of Education. Work stress was measured with a standardized evaluation scale of the effort-reward imbalance whose score determined three levels: light (<1), moderate (from 1 to 1.5) and heavy (>1.5). Self-rated health status was measured and computed as a binary variable to define a good general health (score 1–4) vs. a poor one (score 5–8)*.

### Risks of Sickness Absences at Follow-Up

#### Short Sickness Absences

In univariable analysis, all the categories of cannabis use, compared to never used, were positively associated with short sickness absences (Table 2). In all multivariable analyses, cannabis use more than once a month remained significantly associated with an increased risk of short sickness absences [OR = 1.56 (95% CI:1.32,1.83) for the fully-adjusted model] (Table 2). This association remained also significant in all strata after stratifying for age, gender and smoking status (Supplementary Tables).

**Table 2:** Associations between the frequency of cannabis use at inclusion and the risk of sickness absences at one-year (*N* = 87,273).

		**Model 1**		**Model 2**		**Model 3**		**Model 4**	
		**Univariable**		**Adjusted for age and gender**		**Adjusted for age, gender and tobacco consumption**		**Adjusted for all the covariables**	
	**(F)**	**OR (95% IC)**	***p*-value**	**OR (95% IC)**	***p*-value**	**OR (95% IC)**	***p-*value**	**OR (95% IC)**	***p*-value**
Short sickness absences (<7 days)	(1)	–		–		–		–	
	(2)	1.15 (1.09, 1.21)	<0.001	1.10 (1.04, 1.16)	<0.001	1.02 (0.97, 1.08)	0.4	1.11 (1.05, 1.17)	<0.001
	(3)	1.27 (1.04, 1.53)	0.017	1.14 (0.93, 1.38)	0.2	1.05 (0.86, 1.28)	0.6	1.18 (0.96, 1.43)	0.11
	(4)	1.86 (1.58, 2.16)	<0.001	1.67 (1.42, 1.96)	<0.001	1.51 (1.28, 1.76)	<0.001	1.56 (1.32, 1.83)	<0.001
Medium sickness absences (7–28 days)	(1)	–		–		–		–	
	(2)	0.97 (0.92, 1.02)	0.3	0.98 (0.92, 1.03)	0.4	0.89 (0.84, 0.94)	<0.001	1.00 (0.94, 1.06)	0.9
	(3)	0.96 (0.77, 1.19)	0.7	0.96 (0.77, 1.19)	0.7	0.87 (0.69, 1.07)	0.2	1.02 (0.81, 1.26)	0.9
	(4)	1.39 (1.16, 1.64)	<0.001	1.41 (1.18, 1.68)	<0.001	1.23 (1.02, 1.46)	0.025	1.29 (1.07, 1.54)	0.007
Long sickness absences (>28 days)	(1)	–		–		–		–	
	(2)	0.88 (0.82, 0.94)	<0.001	0.94 (0.88, 1.01)	0.084	0.82 (0.76, 0.88)	<0.001	0.94 (0.88, 1.02)	0.12
	(3)	0.89 (0.67, 1.16)	0.4	1.00 (0.75, 1.31)	0.9	0.86 (0.65, 1.12)	0.3	1.04 (0.77, 1.37)	0.8
	(4)	1.19 (0.95, 1.48)	0.12	1.39 (1.10, 1.73)	0.004	1.14 (0.90, 1.42)	0.3	1.18 (0.93, 1.48)	0.2

*(F) Frequency of cannabis use: (1) never used, (2) no use in the last 12 months, (3) less than once a month in the last year, (4) more than once a month in the last year*.

There were interactions between cannabis use and gender (*p* = 0.039) and self-rated health (*p* = 0.043). Men were at higher risk of sickness absence compared to women (Supplementary Tables). Associations remained significant only among those with better health status (Supplementary Tables).

**Figure 2:**
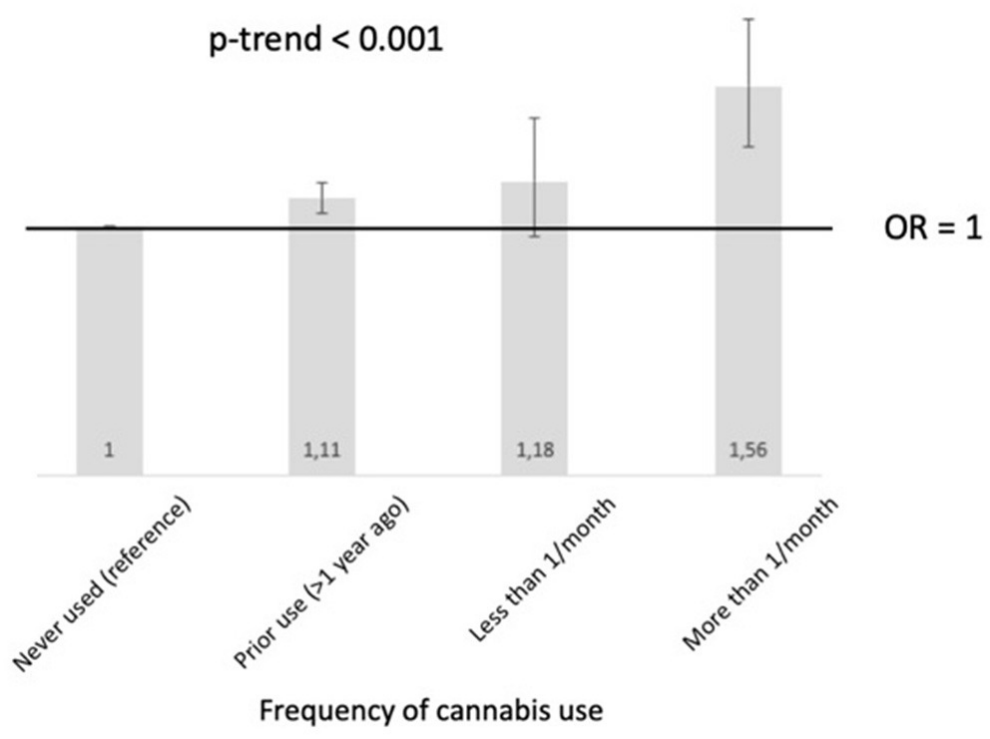
Representation of the dose-dependent relationship between cannabis use at inclusion and the risk of short sickness absence at one year after adjustment for sociodemographic factors, chronic conditions, and occupational factors (*N* = 87,273).

There was a dose-dependent relationship between cannabis use and the risk of short sickness absences at one-year [OR = 1.13 (95% CI:1.08,1.18), *p*-for-trend <0,001 for the fully-adjusted model] (Figure 2).

#### Medium Sickness Absences

In univariable analysis, cannabis use more than once a month was positively associated with medium sickness absences (Table 2). In the model adjusted for age, gender and tobacco consumption, prior cannabis use more than one year ago was negatively associated with medium sickness absences, this result being no longer observed in the other models (Table 2). In all multivariable analyses, cannabis use more than once a month remained significantly associated with an increased risk of medium sickness absences [OR = 1.29 (95% CI:1.07,1.54) for the fully-adjusted model] (Table 2). After stratifications for gender, age and smoking status, this association remained significant in the oldest participants [OR = 3.06 (95% CI:1.62,5.41)] and in men [OR = 1.40 (95% CI:1.12,1.75)] (Supplementary Tables).

There were interactions for age (*p* < 0.001), tobacco consumption (*p* = 0.024), marital status (*p* = 0.023) and type of work contract (*p* = 0.003). After stratifying for marital status and type of work contract, cannabis use remained significantly associated with medium sickness absences in the following strata: married or in a civil partnership participants, separated, divorced or widowed participants, and open-ended contract (Supplementary Tables).

#### Long Sickness Absences

In univariable analysis, prior cannabis use more than one year ago was negatively associated with long sickness absences (Table 2). In the model adjusted for age and gender, cannabis use more than once a month was positively associated with long sickness absence [OR = 1.39 (95% CI:1.10,1.73)] (Table 2). When adjusting additionally for tobacco consumption, this association was no longer significant but prior cannabis use more than one year ago was again negatively associated with long sickness absences (Table 2). However, there were no significant associations between cannabis use and long sickness absences in the fully-adjusted model (Table 2).

After stratifications for age, gender and smoking status, cannabis use more than once a month was positively associated with long sickness absences in participants aged between 35 and 50 [OR = 1.59 (95% CI:1.11,2.20)] and men [OR = 1.58 (95% CI:1.19,2.07)], with a significant interaction for gender (*p* = 0.035) and tobacco consumption (*p* = 0.035) (Supplementary Tables).

### Sensitivity Analyses

In sensitivity analyses, positive associations between cannabis use more than once a month and short sickness absences at one-year of follow-up remained significant for participants without any prior sickness absence in the last 3 years (Supplementary Tables). Moreover, positive associations with cannabis use more than once a month remained significant for those with at least one prior sickness absence regarding the risk of medium sickness absences at one-year (Supplementary Tables). In addition, positive association between short sickness absences and prior cannabis use more than one year ago was significant for both strata (Supplementary Tables).

When considering sickness absences as a count variable rather than a binary one, all the associations from the fully-adjusted models remained significant and with similar effect sizes (Supplementary Tables). Finally, compared to participants who didn't report their cannabis use, included participants were more likely to be women, older, in couple, from higher occupational grade, with higher income, with a less number of pack-years, with a better self-rated health status, with less comorbidities, with open-ended contract and with less work stress (Supplementary Tables).

## Discussion

Our main objective was to examine the prospective associations between cannabis use and the risk of short, medium and long sickness absences at one-year of follow-up in a large population-based cohort. We found that cannabis use more than once a month was associated with an increased risk of subsequent short and medium sickness absences, even after adjusting for sociodemographic, chronic conditions and occupational factors. Dose-dependent relationships were also found regarding short sickness absences. Stratified analyses showed that these associations may be more prevalent among older participants, men, and those with a good self-rated health, living with a partner, and having an open-ended work contract (22–24).

To our knowledge, this is the first study to examine the prospective associations of cannabis use and sickness absences in a wide national population-based cohort with participants from various sociodemographic and occupational backgrounds. The systematic linkage with administrative medical registries provides an assessment of outcomes based on objective and exhaustive data. Moreover, thanks to the large sample, we have sufficient statistical power to take into account a great number of potential confounders and to examine further moderating effects. This study has also limitations. First, although CONSTANCES participants were recruited randomly, participants in epidemiological cohorts usually differ from the general population (25). Thus, results should be extrapolated to other settings with caution. Second, only 89.9% of participants in the CONSTANCES cohort reported their frequency of cannabis use at baseline. As expected, this population tend to be healthier and with higher socioeconomic conditions. However, the objective of the study was to study the associations between two variables and not to obtain prevalence data and such non-response bias should result in an underestimation of our associations. Third, the frequency of cannabis use was measured at inclusion as an overall measure of the frequency of cannabis use in the past 12 months, making it impossible to take into account for the roles of past exposures or changes in consumption during this period. Fourth, a complete history of sickness absences was not collected. Nevertheless, these data were available since 2009, i.e., 3 years before the inclusion of the first participants in the cohort and sensitivity analyses showed that the associations remained significant in the subgroup of participants who had no prior sickness absences in the 3 years preceding inclusion. Finally, some potential confounding factors were not measured, such as personality and social environment.

Overall, our results are in accordance with our a priori hypotheses as we found evidence that consuming cannabis increased the risk of sickness absences. Our results were primarily concerned with the risk of short sickness absences. The acute effects of cannabis, such as reduced cognitive performance (attention, concentration, planning, working memory), symptoms of intoxication (mood swings and disinhibition or, conversely, distrust and feelings of persecution) and withdrawal symptoms (irritability, anxiety, asthenia) could be particularly harmful to the ability to fulfill professional obligations and lead to short sickness absences. As cannabis is an illicit substance whose use is punishable by law in France, some people showing visible signs of use might consider taking a short sickness absence to avoid professional misconduct. The chronic effects of cannabis such as induced psychiatric disorders (mood disorders, anxiety disorders) and somatic pathologies (cancers, cardiovascular diseases) require long-term medical treatment and could therefore lead to longer sickness absences. Regarding the stronger associations in men, they tend to consume larger quantities of cannabis than women and its use is more frequently accompanied by other behaviors harmful to health (alcohol, tobacco), leading to an increase in risks and damage after a certain duration of exposure (1, 26). Moreover, short sickness absences affect more people with a good self-rated health, who may be more inclined to risk behaviors, and therefore to their consequences, including on work (23). On the other hand, people with a bad self-rated health, perhaps already caused by substance use, may use less to maintain their health, which has less impact on their work. They may also have other risk factors for sickness absences that may have a greater role than cannabis use. The duration of cannabis exposure is likely to be longer in older individuals, which may increase their risk of developing somatic pathologies related to their cannabis use. In addition, workers with greater financial security (i.e., living with a partner, having an open-ended contract), are likely to have less difficulty accepting a longer sickness absence than those with less financial security, who may be more likely to refuse or not request reimbursement for days of absence (24). As with self-rated health, another hypothesis could be that less favorable socio-economic conditions may already be the consequence of cannabis use.

Associations between cannabis and long sickness absences were only significant in men and in participants aged 35–50 years. One might hypothesize that participants from these groups had a lower likelihood of experiencing a shorter sickness absence during the follow-up period because they were more frequently on long sickness absence. However, subgroups analyses revealed significant associations among men and across all age groups for short sickness absences. Long sickness absences tend to occur in sicker people, suggesting that cannabis is not the major factor involved for this outcome, especially after the age of 50 (27). The confounding effect of smoking is potentially partially captured, at least for some diseases, by the adjustment for pack-years, hence the disappearance of significance for heavy cannabis users after the introduction of this covariable in the model. Furthermore, some individuals may use cannabis for medical conditions (e.g., back pain, headaches, sleep disorders) (28–30).

In conclusion, the present study showed that cannabis use more than once a month was associated with an increased risk of subsequent short and medium sickness absences, even after adjusting for sociodemographic factors, chronic conditions and occupational factors, and with dose-dependent relationships regarding short sickness absences. These findings could be used in prevention public health campaigns to better inform on the harm associated with cannabis use. The innovative nature of this awareness-raising message, which focuses on occupational damage of cannabis use, could constitute an important motivational tool for workers to reduce their cannabis use. Moreover, sickness absences are linked to an increase in morbidity and mortality, partially in relation with a deterioration in health behaviors during these absences. Thus, preventing from detrimental health behaviors that could promote sickness absences could be of paramount interest, because such detrimental behaviors tend to increase while being in sickness absence (11). From a public health perspective, it is therefore essential to prevent the entry into this vicious circle. Future longitudinal studies with repeated exposure measurements could better document the possible causal links between cannabis and sickness absences. In addition, information on medical motives and the type of doctor who prescribed sickness absences (e.g., GP, Emergency physician, psychiatrist) would be helpful to improve our understanding of the mechanisms underlying these associations. Future research should also attempt to better understand, for example with qualitative studies, the motivations of the working population to use cannabis and to explore the psychosocial factors involved in the links between cannabis and sickness absences.

## Data Availability Statement

The datasets presented in this article are not readily available because personal health data underlying the findings of our study are not publicly available due to legal reasons related to data privacy protection. However, the data are available upon request to all interested researchers after authorization of the French “Commission Nationale de l'Informatique et des Libertés”. Requests to access the datasets should be directed to MZ (rf.mresni@sniz.eiram) and MG (rf.mresni@grebdlog.lecram).

## Ethics Statement

The studies involving human participants were reviewed and approved by National Data Protection Authority (Commission Nationale de l'Informatique et des Libertés—CNIL, No. 910486) and Institutional Review Board of the National Institute for Medical Research—INSERM (No. 01–011). The patients/participants provided their written informed consent to participate in this study.

## Author Contributions

AD: conceptualization, methodology, software, and writing—original draft. GA: conceptualization, methodology, writing—review and editing, resources, supervision, and validation. MZ: writing—review and editing, project administration, data curation, resources, supervision, and validation. YR and CL: writing—review and editing, supervision, and validation. MG: writing—review and editing, project administration, data curation, supervision, and validation. AL: methodology, writing—review and editing, supervision, and validation. All authors contributed to the article and approved the submitted version.

## Conflict of Interest

GA declares consultant and/or speaker fees from Pfizer, Zentiva, Pierre Fabre, and Lundbeck. CL declares consultant and/or speaker fees from Boehringer Ingelheim, Janssen-Cilag, Lundbeck, and Otsuka Pharmaceutical outside the submitted work. The CONSTANCES cohort benefits from grant ANR-11-INBS-0002 from the French National Research Agency. CONSTANCES was supported by the Caisse Nationale d'Assurance Maladie, the French Ministry of Health, the Ministry of Research, the Institut National de la Sante et de la Recherche Medicale (INSERM). CONSTANCES is also partly funded by AstraZeneca, Lundbeck, L'Oréal, and Merck Sharp & Dohme Corp managed by INSERM-Transfert. This study was supported by the MILDECA (Interministerial Mission for Combating Drugs and Addictive Behaviors). The funders had no role in the design and conduct of the study, collection, management, analysis, and interpretation of the data, preparation, review, or approval of the manuscript, and decision to submit the manuscript for publication. The remaining authors declare that the research was conducted in the absence of any commercial or financial relationships that could be construed as a potential conflict of interest.

## Publisher's Note

All claims expressed in this article are solely those of the authors and do not necessarily represent those of their affiliated organizations, or those of the publisher, the editors and the reviewers. Any product that may be evaluated in this article, or claim that may be made by its manufacturer, is not guaranteed or endorsed by the publisher.
