# Supplementary material for: Cannabis Use Increases the Risk of Sickness Absence: Longitudinal Analyses From the CONSTANCES Cohort
Source: Front Public Health. 2022 May 30;10:869051. doi: 10.3389/fpubh.2022.869051 (PMC9197417; doi:10.3389/fpubh.2022.869051)
Supplement: Supplementary file 1 [file Table_1.DOCX]

**Supplemental Tables**

**1.**

|  |  | *Stratification on age* | | | | | |
| --- | --- | --- | --- | --- | --- | --- | --- |
|  |  | 18-35 years old | | 35-50 years old | | 50-65 years old | |
|  | Frequency of cannabis use | OR  (95% IC) | p-value | OR  (95% IC) | p-value | OR  (95% IC) | p-value |
| Short sickness absences  (<7 days)  N=6 771 | (1) | - |  | - |  | - |  |
|  | (2) | 0.99  (0.90, 1.09) | 0.8 | 1.15  (1.06, 1.25) | <0.001 | 1.20  (1.05, 1.35) | 0.005 |
|  | (3) | 1.07  (0.81, 1.40) | 0.6 | 1.20  (0.85, 1.64) | 0.3 | 1.81  (0.83, 3.50) | 0.1 |
|  | (4) | 1.41  (1.12, 1.76) | 0.003 | 1.69  (1.29, 2.18) | <0.001 | 2.37  (1.17, 4.38) | 0.010 |
| Medium sickness absences (7-28 days)  N=6 370 | (1) | - |  | - |  | - |  |
|  | (2) | 0.88  (0.79, 0.99) | 0.027 | 1.05  (0.97, 1.15) | 0.2 | 1.01  (0.89, 1.15) | 0.8 |
|  | (3) | 0.72  (0.50, 1.01) | 0.065 | 1.34  (0.96, 1.83) | 0.076 | 1.67  (0.76, 3.24) | 0.2 |
|  | (4) | 1.21  (0.93, 1.56) | 0.14 | 1.26  (0.93, 1.68) | 0.13 | 3.06  (1.62, 5.41) | <0.001 |
| Long sickness absences (>28 days)  N=4 046 | (1) | - |  | - |  | - |  |
|  | (2) | 0.99  (0.85, 1.14) | 0.8 | 0.92  (0.82, 1.02) | 0.13 | 0.90  (0.78, 1.05) | 0.2 |
|  | (3) | 1.05  (0.68, 1.55) | 0.8 | 1.14  (0.72, 1.72) | 0.5 | 0.84  (0.25, 2.09) | 0.7 |
|  | (4) | 0.97  (0.66, 1.37) | 0.9 | 1.59  (1.11, 2.20) | 0.008 | 1.72  (0.70, 3.60) | 0.2 |

**Stratification on age**
