# Supplementary material for: Cannabis Use Increases the Risk of Sickness Absence: Longitudinal Analyses From the CONSTANCES Cohort
Source: Front Public Health. 2022 May 30;10:869051. doi: 10.3389/fpubh.2022.869051 (PMC9197417; doi:10.3389/fpubh.2022.869051)
Supplement: Supplementary file 2 [file Table_2.DOCX]

**Supplemental Tables**

**2. Stratification on gender**

|  |  | *Stratification on gender* | | | |
| --- | --- | --- | --- | --- | --- |
|  |  | Men | | Women | |
|  | Frequency of cannabis use | OR  (95% IC) | p-value | OR  (95% IC) | p-value |
| **Short sickness absences**  **(<7 days)  N=6 771** | (1) | - |  | - |  |
|  | (2) | 1.06  (0.98, 1.15) | 0.2 | 1.13  (1.05, 1.22) | 0.001 |
|  | (3) | 1.25  (0.96, 1.60) | 0.095 | 1.11  (0.80, 1.51) | 0.5 |
|  | (4) | 1.65  (1.36, 2.00) | <0.001 | 1.37  (1.00, 1.85) | 0.043 |
| **Medium sickness absences (7-28 days)  N=6 370** | (1) | - |  | - |  |
|  | (2) | 0.99  (0.90, 1.08) | 0.8 | 1.00  (0.92, 1.08) | 0.9 |
|  | (3) | 1.18  (0.87, 1.57) | 0.3 | 0.89  (0.62, 1.24) | 0.5 |
|  | (4) | 1.40  (1.12, 1.75) | 0.003 | 1.20  (0.85, 1.64) | 0.3 |
| **Long sickness absences (>28 days)  N=4 046** | (1) | - |  | - |  |
|  | (2) | 0.97  (0.86, 1.09) | 0.6 | 0.93  (0.84, 1.02) | 0.13 |
|  | (3) | 1.02  (0.66, 1.51) | 0.9 | 1.13  (0.75, 1.65) | 0.5 |
|  | (4) | 1.58  (1.19, 2.07) | 0.001 | 0.69  (0.40, 1.09) | 0.14 |
