# Supplementary material for: Cannabis Use Increases the Risk of Sickness Absence: Longitudinal Analyses From the CONSTANCES Cohort
Source: Front Public Health. 2022 May 30;10:869051. doi: 10.3389/fpubh.2022.869051 (PMC9197417; doi:10.3389/fpubh.2022.869051)
Supplement: Supplementary file 3 [file Table_3.DOCX]

**Supplemental Tables**

**3. Stratification on smoking status**

|  |  | Smokers | | Non smokers | |
| --- | --- | --- | --- | --- | --- |
|  | Frequency of cannabis use | OR  (95% IC) | p-value | OR  (95% IC) | p-value |
| **Short sickness absences**  **(<7 days)  N=6 771** | (1) | - |  | - |  |
|  | (2) | 1.07  (0.94, 1.22) | 0.3 | 1.14  (1.07, 1.21) | <0.001 |
|  | (3) | 0.99  (0.73, 1.32) | 0.9 | 1.40  (1.05, 1.84) | 0.020 |
|  | (4) | 1.49 (1.21, 1.84) | <0.001 | 1.51  (1.00, 2.18) | 0.038 |
| **Medium sickness absences (7-28 days)  N=6 370** | (1) | - |  | - |  |
|  | (2) | 0.88  (0.77, 1.00) | 0.059 | 1.08  (1.01, 1.16) | 0.017 |
|  | (3) | 1.01  (0.74, 1.35) | 0.9 | 0.95  (0.65, 1.35) | 0.8 |
|  | (4) | 1.19  (0.94, 1.49) | 0.14 | 1.36  (0.85, 2.05) | 0.2 |
| **Long sickness absences (>28 days)  N=4 046** | (1) | - |  | - |  |
|  | (2) | 0.94  (0.80, 1.10) | 0.4 | 1.02  (0.93, 1.10) | 0.7 |
|  | (3) | 0.92  (0.61, 1.32) | 0.7 | 1.21  (0.76, 1.84) | 0.4 |
|  | (4) | 1.03  (0.77, 1.37) | 0.8 | 1.32  (0.69, 2.27) | 0.4 |
