# Supplementary material for: Cannabis Use Increases the Risk of Sickness Absence: Longitudinal Analyses From the CONSTANCES Cohort
Source: Front Public Health. 2022 May 30;10:869051. doi: 10.3389/fpubh.2022.869051 (PMC9197417; doi:10.3389/fpubh.2022.869051)
Supplement: Supplementary file 4 [file Table_4.DOCX]

**Supplemental Tables**

**4. Stratification on marital status**

|  |  | *Stratification on marital status* | | | | | |
| --- | --- | --- | --- | --- | --- | --- | --- |
|  |  | Single | | Married or in a civil partnership | | Separated, divorced or widowed | |
|  | Frequency of cannabis use | OR  (95% IC) | p-value | OR  (95% IC) | p-value | OR  (95% IC) | p-value |
| **Medium sickness absences (7-28 days)  N=6 370** | (1) | - |  | - |  | - |  |
|  | (2) | 0.91  (0.82, 1.01) | 0.077 | 1.04  (0.97, 1.13) | 0.3 | 1.00  (0.84, 1.19) | 0.9 |
|  | (3) | 1.01  (0.73, 1.37) | 0.9 | 0.91  (0.61, 1.29) | 0.6 | 1.60  (0.80, 2.94) | 0.2 |
|  | (4) | 1.07  (0.82, 1.37) | 0.6 | 1.52  (1.12, 2.01) | 0.005 | 1.88  (1.02, 3.26) | 0.032 |
