# Supplementary material for: Cannabis Use Increases the Risk of Sickness Absence: Longitudinal Analyses From the CONSTANCES Cohort
Source: Front Public Health. 2022 May 30;10:869051. doi: 10.3389/fpubh.2022.869051 (PMC9197417; doi:10.3389/fpubh.2022.869051)
Supplement: Supplementary file 5 [file Table_5.DOCX]

**Supplemental Tables**

**5. Stratification on self-rated health**

|  |  | *Stratification on self-rated health* | | | |
| --- | --- | --- | --- | --- | --- |
|  |  | Good | | Bad | |
|  | Frequency of cannabis use | OR  (95% IC) | p-value | OR  (95% IC) | p-value |
| Short sickness absences (<7 days)  N=6 771 | (1) | - |  | - |  |
|  | (2) | 1.14  (1.07, 1.21) | <0.001 | 0.93  (0.80, 1.07) | 0.3 |
|  | (3) | 1.25  (1.01, 1.54) | 0.039 | 0.81  (0.43, 1.42) | 0.5 |
|  | (4) | 1.64  (1.37, 1.95) | <0.001 | 1.17  (0.77, 1.74) | 0.4 |
