# Supplementary material for: Cannabis Use Increases the Risk of Sickness Absence: Longitudinal Analyses From the CONSTANCES Cohort
Source: Front Public Health. 2022 May 30;10:869051. doi: 10.3389/fpubh.2022.869051 (PMC9197417; doi:10.3389/fpubh.2022.869051)
Supplement: Supplementary file 6 [file Table_6.DOCX]

**Supplemental Tables**

**6. Stratification on type of work contract**

|  |  | *Stratification on type of work contract* | | | |
| --- | --- | --- | --- | --- | --- |
|  |  | Fixed-term contract | | Open-ended contract | |
|  | Frequency of cannabis use | OR  (95% IC) | p-value | OR  (95% IC) | p-value |
| **Medium sickness absences (7-28 days)  N=6 370** | (1) | - |  | - |  |
|  | (2) | 1.01  (0.75, 1.35) | 0.9 | 1.00  (0.94, 1.06) | 0.9 |
|  | (3) | 0.00  (0.00, 0.00) | 0.9 | 1.10  (0.87, 1.37) | 0.4 |
|  | (4) | 0.89  (0.38, 1.81) | 0.8 | 1.32  (1.09, 1.59) | 0.003 |
