# Supplementary material for: Cannabis Use Increases the Risk of Sickness Absence: Longitudinal Analyses From the CONSTANCES Cohort
Source: Front Public Health. 2022 May 30;10:869051. doi: 10.3389/fpubh.2022.869051 (PMC9197417; doi:10.3389/fpubh.2022.869051)
Supplement: Supplementary file 7 [file Table_7.DOCX]

**Supplemental Tables**

**7. Stratification for sickness absences in the three years before inclusion**

|  |  | *Stratification for sickness absences in the three years before inclusion* | | | |
| --- | --- | --- | --- | --- | --- |
|  |  | 0 sickness absence | | >1 sickness absence | |
|  | Frequency of cannabis use | OR  (95% IC) | p-value | OR  (95% IC) | p-value |
| Short sickness absences  (<7 days)  N=6 771 | (1) | - |  | - |  |
|  | (2) | 1.08  (1.02, 1.15) | 0.013 | 1.23  (1.05, 1.44) | 0.012 |
|  | (3) | 1.18  (0.95, 1.45) | 0.14 | 1.06  (0.55, 1.96) | 0.9 |
|  | (4) | 1.51  (1.26, 1.80) | <0.001 | 1.60  (0.99, 2.59) | 0.054 |
| Medium sickness absences (7-28 days)  N=6 370 | (1) | - |  | - |  |
|  | (2) | 1.01  (0.94, 1.07) | 0.9 | 0.88  (0.74, 1.04) | 0.13 |
|  | (3) | 0.94  (0.73, 1.19) | 0.6 | 1.57  (0.84, 2.87) | 0.15 |
|  | (4) | 1.17  (0.95, 1.43) | 0.12 | 1.82  (1.11, 2.94) | 0.016 |
| Long sickness absences (>28 days)  N=4 046 | (1) | - |  | - |  |
|  | (2) | 0.94  (0.87, 1.02) | 0.2 | 0.89  (0.74, 1.07) | 0.2 |
|  | (3) | 1.05  (0.76, 1.41) | 0.8 | 0.95  (0.43, 1.92) | 0.9 |
|  | (4) | 1.24  (0.95, 1.58) | 0.1 | 0.75  (0.40, 1.34) | 0.4 |
