# Supplementary material for: Cannabis Use Increases the Risk of Sickness Absence: Longitudinal Analyses From the CONSTANCES Cohort
Source: Front Public Health. 2022 May 30;10:869051. doi: 10.3389/fpubh.2022.869051 (PMC9197417; doi:10.3389/fpubh.2022.869051)
Supplement: Supplementary file 8 [file Table_8.DOCX]

**Supplemental Tables**

**8. Negative binomial regression**

|  |  | *Negative binomial regression* | |
| --- | --- | --- | --- |
|  | Frequency of cannabis use | OR  (95% IC) | p-value |
| **Short sickness absences**  **(<7 days)  N=6 771** | (1) | - |  |
|  | (2) | 1.15  (1.09, 1.22) | <0.001 |
|  | (3) | 1.26  (1.03, 1.54) | 0.022 |
|  | (4) | 1.50  (1.26, 1.78) | <0.001 |
| **Medium sickness absences (7-28 days)  N=6 370** | (1) | - |  |
|  | (2) | 1.00  (0.94, 1.06) | 0.9 |
|  | (3) | 0.98  (0.78, 1.22) | 0.9 |
|  | (4) | 1.23  (1.02, 1.47) | 0.027 |
| **Long sickness absences (>28 days)  N=4 046** | (1) | - |  |
|  | (2) | 0.97  (0.90, 1.05) | 0.4 |
|  | (3) | 0.98  (0.73, 1.30) | 0.9 |
|  | (4) | 1.25  (0.99, 1.57) | 0.052 |
