# Supplementary material for: Cannabis Use Increases the Risk of Sickness Absence: Longitudinal Analyses From the CONSTANCES Cohort
Source: Front Public Health. 2022 May 30;10:869051. doi: 10.3389/fpubh.2022.869051 (PMC9197417; doi:10.3389/fpubh.2022.869051)
Supplement: Supplementary file 9 [file Table_9.DOCX]

**Supplemental Tables**

|  | **Study population** | **Not reported frequency of cannabis use** | **p-value**  **(χ^2^ test or t-test)** |
| --- | --- | --- | --- |
|  | **(N=87,273)** | **(N=9,995)** |  |
| **Gender** |  |  | p<0.001 |
| Men | 39762 (45.6%) | 5413 (54.2%) |  |
| Women | 47511 (54.4%) | 4582 (45.8%) |  |
| **Age** |  |  | p<0.001 |
| Median [Min, Max] | 43.5 [18.5, 65.0] | 39.0 [31.0, 49.0] |  |
| [18,35] | 21361 (24.5%) | 3884 (38.9%) |  |
| (35,50] | 41000 (47.0%) | 3852 (38.5%) |  |
| (50,65] | 24912 (28.5%) | 2259 (22.6%) |  |
| **Frequency of cannabis use** |  |  | NA |
| Never used | 52540 (60.2%) | NA |  |
| Prior use more than one year ago | 31975 (36.6%) | NA |  |
| Less than once a month | 1272 (1.5%) | NA |  |
| More than once a month | 1486 (1.7%) | NA |  |
| **Marital status** |  |  | p<0.001 |
| Single | 23951 (27.4%) | 4296 (43.0%) |  |
| Married or in a civil partnership | 54067 (62.0%) | 4670 (46.7%) |  |
| Separated, divorced or widowed | 9255 (10.6%) | 1029 (10.3%) |  |
| **Occupational grade** |  |  | p<0.001 |
| Blue collar worker and clerk | 29923 (34.3%) | 3970 (39.7%) |  |
| Intermediate worker | 26719 (30.6%) | 2589 (25.9%) |  |
| Executive | 30631 (35.1%) | 3436 (34.4%) |  |
| **Income** |  |  | p<0.001 |
| <1500€/month | 5598 (6.4%) | 1278 (12.8%) |  |
| 1500-4200€/month | 52457 (60.1%) | 6225 (62.3%) |  |
| >4200€/month | 29218 (33.5%) | 2492 (24.9%) |  |
| **Education** |  |  | p<0.001 |
| ISCED - levels 0-4 | 27911 (32.0%) | 3589 (35.9%) |  |
| ISCED - levels 5-6 | 34076 (39.0%) | 3296 (33.0%) |  |
| ISCED - levels 7-8 | 25286 (29.0%) | 3110 (31.1%) |  |
| **Number of pack-years (10 PY)** |  |  | p<0.001 |
| Median [Min, Max] | 0 [0, 120] | 3 [0, 10] |  |
| **Self-rated health** |  |  | p<0.001 |
| Good | 77743 (89.1%) | 8737 (87.4%) |  |
| Bad | 9530 (10.9%) | 1258 (12.6%) |  |
| **History of depression** |  |  | p=0.17 |
| No | 75313 (86.3%) | 8575 (85.8%) |  |
| Yes | 11960 (13.7%) | 1420 (14.2%) |  |
| **Chronic condition** |  |  | p=0.04 |
| No | 2795 (81.7%) | 281 (77.2%) |  |
| Yes | 626 (18.3%) | 83 (22.8%) |  |
| **Work contract** |  |  | p<0.001 |
| Open-ended | 82374 (94.4%) | 8891 (88.9%) |  |
| Fixed-term | 4899 (5.6%) | 886 (8.9%) |  |
| Other | NA | 218 (2.2%) |  |
| **Work stress** |  |  | p<0.001 |
| Light | 44757 (51.3%) | 5597 (56.0%) |  |
| Moderate | 30991 (35.5%) | 3197 (32.0%) |  |
| Heavy | 11525 (13.2%) | 1201 (12.0%) |  |
| **Stressful exposure to the public** |  |  | p=0.27 |
| No exposure | 25807 (29.6%) | 3010 (30.1%) |  |
| No stressful exposure | 43228 (49.5%) | 4865 (48.7%) |  |
| Stressful exposure | 18238 (20.9%) | 2120 (21.2%) |  |

**9. Characteristics of the study population and the participants who didn’t report their frequency of cannabis use**
