# Supplementary material for: Cannabis Use Increases the Risk of Sickness Absence: Longitudinal Analyses From the CONSTANCES Cohort
Source: Front Public Health. 2022 May 30;10:869051. doi: 10.3389/fpubh.2022.869051 (PMC9197417; doi:10.3389/fpubh.2022.869051)
Supplement: Supplementary file 10 [file Table_10.DOCX]

**Supplemental Tables**

|  | **At least one short sickness absence** | **At least one medium sickness absence** | **At least one long sickness absence** |
| --- | --- | --- | --- |
|  | **(N=6,771)** | **(N=6,370)** | **(N=4,046)** |
| **Gender** |  |  |  |
| Men | 2977 (44.0%) | 2593 (40.7%) | 1568 (38.8%) |
| Women | 3794 (56.0%) | 3777 (59.3%) | 2478 (61.2%) |
| **Age** |  |  |  |
| Median [Min, Max] | 41.5 [19.5, 65.0] | 43.0 [19.0, 65.0] | 44.5 [18.5, 65.0] |
| [18,35] | 2085 (30.8%) | 1762 (27.7%) | 986 (24.4%) |
| (35,50] | 3043 (44.9%) | 2816 (44.2%) | 1714 (42.4%) |
| (50,65] | 1643 (24.3%) | 1792 (28.1%) | 1446 (33.3%) |
| **Frequency of cannabis use** |  |  |  |
| Never used | 3823 (56.5%) | 3853 (60.5%) | 2542 (62.8%) |
| Prior use more than one year ago | 2644 (39.0%) | 2280 (35.8%) | 1364 (33.7%) |
| Less than once a month | 115 (1.7%) | 90 (1.4%) | 55 (1.4%) |
| More than once a month | 189 (2.8%) | 147 (2.3%) | 85 (2.1%) |
| **Marital status** |  |  |  |
| Single | 2109 (31.1%) | 1820 (28.6%) | 1061 (26.2%) |
| Married or in a civil partnership | 3913 (57.8%) | 3754 (58.9%) | 2380 (58.8%) |
| Separated, divorced or widowed | 749 (11.1%) | 796 (12.5%) | 605 (15.0%) |
| **Occupational grade** |  |  |  |
| Blue collar worker and clerk | 3415 (50.4%) | 3577 (56.2%) | 2483 (61.4%) |
| Intermediate worker | 1484 (21.9%) | 1358 (21.3%) | 847 (20.9%) |
| Executive | 1872 (27.6%) | 1435 (22.5%) | 716 (17.7%) |
| **Income** |  |  |  |
| <1500€/month | 623 (9.2%) | 694 (10.9%) | 545 (13.5%) |
| 1500-4200€/month | 4578 (67.6%) | 4388 (68.9%) | 2840 (70.2%) |
| >4200€/month | 1570 (23.2%) | 1288 (20.2%) | 661 (16.3%) |
| **Education** |  |  |  |
| ISCED - levels 0-4 | 2895 (42.8%) | 3207 (50.3%) | 2285 (56.5%) |
| ISCED - levels 5-6 | 2464 (36.4%) | 2084 (32.7%) | 1205 (29.8%) |
| ISCED - levels 7-8 | 1412 (20.9%) | 1079 (16.9%) | 556 (13.7%) |
| **Number of pack-years (10 PY)** |  |  |  |
| Median [Min, Max] | 0 [0, 120] | 1.2 [0, 108] | 2.21 [0, 114] |
| **Self-rated health** |  |  |  |
| Good | 5677 (83.8%) | 5164 (81.1%) | 2987 (73.8%) |
| Bad | 1094 (16.2%) | 1206 (18.9%) | 1059 (26.2%) |
| **History of depression** |  |  |  |
| No | 5573 (82.3%) | 5145 (80.8%) | 3040 (75.1%) |
| Yes | 1198 (17.7%) | 1255 (19.2%) | 1006 (24.9%) |
| **Chronic condition** |  |  |  |
| No | 6428 (94.9%) | 5962 (93.6%) | 3678 (90.9%) |
| Yes | 343 (5.1%) | 408 (6.4%) | 369 (9.1%) |
| **Work contract** |  |  |  |
| Open-ended | 6517 (96.2%) | 6135 (96.3%) | 3903 (96.5%) |
| Fixed-term | 254 (3.8%) | 235 (3.7%) | 143 (3.5%) |
| **Work stress** |  |  |  |
| Light | 2943 (43.5%) | 2688 (42.2%) | 1668 (41.2%) |
| Moderate | 2577 (38.1%) | 2389 (37.5%) | 1386 (34.3%) |
| Heavy | 1251 (18.5%) | 1293 (20.3%) | 992 (24.5%) |
| **Stressful exposure to the public** |  |  |  |
| No exposure | 2264 (33.4%) | 1908 (30.0%) | 1132 (28.0%) |
| No stressful exposure | 2916 (43.1%) | 2872 (45.1%) | 1837 (45.4%) |
| Stressful exposure | 1591 (23.5%) | 1590 (25.0%) | 1077 (26.6%) |

**10. Characteristics of the participants according to sickness absence at follow-up**
